# Redesign Me: Virtual Reality Experience of the Line of Life and Its Connection to a Healthier Self

**DOI:** 10.3390/bs9110111

**Published:** 2019-11-05

**Authors:** Iva Georgieva, Georgi V. Georgiev

**Affiliations:** 1 Department of History and Philosophy of Science, The University of Tokyo, Tokyo 153-8902, Japan; ivavgeorgieva@gmail.com; 2 Institute for Advanced Study, Varna 9010, Bulgaria; 3 Center for Ubiquitous Computing, University of Oulu, 90014 Oulu, Finland

**Keywords:** virtual reality, narrative, self, memory, line of life, storytelling, experience, design, trauma, treatment, health

## Abstract

Virtual Reality is used in various ways for creating a storytelling experience. It gives us the opportunity to imagine one’s life events as a story, and in settings that are intended to aid the self, such as treatment of trauma, anxiety, phobia, etc. This paper discusses the ways that challenging experiences change the way people perceive their life narratives and form their memories. This paper suggests that virtual reality (VR) can be used for the exploration of alternative scenarios in order to see one’s overall line of life in a new and healthier way. Considering the theoretical background of the narrative self, this research proposes a novel view of VR immersion as a medium for constructing a new storyline and attitude to the past. The approach would also influence attitudes regarding the present and future, and thus better shape the narrative of the self, which can lead to healthier life experiences.

## 1. Introduction

Virtual reality (VR) presents the opportunity for storytelling experiences [1,2,3,4] and representations of the self [5,6,7,8]; as such, it should also be considered a powerful tool for representing events that comprise people’s memories. The features of VR have been a topic of analysis in various multidisciplinary studies [9,10,11,12,13,14]. This paper considers the philosophical idea of the narrative self [15,16,17], in addition to reviewing studies which have explored the impact of the virtual on the real self [18,19,20], to propose a novel view on the way the line of life is experienced and may be represented in VR. The line of life, which is an understanding of the self, forms as a narrative explanation of the events stored in the memory [21]. It is an abstract and personalized interpretation that can be represented in VR, and thus it can be analyzed. As VR applications are often meant to be used as entertainment [22,23] and as tools for deep narrative experiences, such as in games, it can effectively represent life events. The narrative nature of the self and the storytelling features of VR, as a tool of great flexibility, allow for movement through narratives the way traditional storytelling techniques do [24], thus forming a truly successful approach to a new look upon one’s line of life.

### 1.1. Serious Applications of VR

VR is considered a practical tool in the field of healthcare research for treatment of various conditions, including several interventions for the reconstruction of life narratives. Increasingly, studies are exploring how VR can be used in therapeutic settings as a tool for exposure therapy and recovery [25,26,27]. However, this study focuses on ways of recreating past events and creating novel scenarios. Such exposure, with habituation purposes [28,29], is not the only way that VR can help in the quest for recovery and well-being. VR can also support visualization of one’s memory of events or line of life with the purpose of recovering the self and its healthy narrative. In this way, recognition of patterns in the line of life may be enhanced such that one may gain a better understanding of one’s life. This study also considers how ideas of reality and time are affected by challenging experiences and events that disrupt normal personal narratives.

### 1.2. VR for Training or Treatment

When VR is used for training or treatment [30,31,32,33], it can help a person to see events or emotions in a new light, perceive stories from a different angle, and/or reorder experiences in a more meaningful plotline. Such therapeutic applications of VR are considered to be treatment for serious health conditions (e.g., behavioral problems such as addictions) or to train a person for situations never experienced before (e.g., earthquake preparedness). However, this suggests VR could be applied in other ways, such as cultivating relaxation or offering viewpoints not visible otherwise, on testing problems [24] (Bucher, 2017). A person’s perception of reality and self-identity can change drastically in response to challenging events [34,35]. VR can represent such events from a more objective [36], or at least observational, perspective [37], and with the help of a therapist it can reveal unconscious patterns and revise unhealthy perceptions.

The capacity of VR to help in the visualization of personal narratives and the creation of new stories within the original context is the background of this study [38]. The study explores the ways VR can aid in redesigning the line of life, helping the self-narrative, providing a different perspective, changing the perception of events, and reconstructing the line of life in a healthier way.

## 2. Philosophical Background: The Self-Narrative Concept and the Line of Life

### 2.1. Reexperience and Recreation of the Line of Life

This study explored the prospects for using VR as a medium to recreate one’s life story [38]. Drawing on the concept that self-narrative constitutes one’s personal identity or self, recreation of the line of life through challenging situations can lead to an overall recreational effect on the self. As mentioned above, traumatic events change the way people perceive reality. Moreover, their memory is affected such that they become locked in the past and cannot envision their future [39,40]. People so affected can benefit from VR experiences when guided by a therapist, and specifically, they may regain a more objective view of their life story through the immersion and by construction of a healthier narrative meaning and explanation of their memories of key events [41,42,43]. This paper explores ways of visualizing one’s life narrative in VR and specific methods of designing experiences for self-recreation.

In comparison to other studies, here the authors emphasize the role of the medium. VR provides the opportunity to present an individual with a number of options with a view to obtaining resolution and adding meaning to a life story through visualization of the memory of past events and imagining the overall line of life in a consistent and healthy way, rather than a damaged and broken one. In this sense, its contribution is in the quest for clearly sought story meaning and novel narration of the line of life, aiding in the search for meaning in the worst events and in personal evolution. This proposition is distinct from prior works on life representation in VR [44] or immersion effects on the self [45], and is also distinct from initiatives which do not consider the storytelling role of VR in the context of narrative therapy [46,47].

### 2.2. Self-Narrative and Line of Life

A philosophical account of human life regards us as narrative-generating machines, assuming the self to be the protagonist of such storylines [17]. Having this in mind, cases of psychological trauma may be regarded as a break in one’s narrative line of life [38]. The self-narrative is used to attain meaning, and attaching it to life events often requires rearrangement of the life events according to their personal significance. Negative events may be of considerable significance, and if one has the strength to overcome them, they can lead to growth and become just as significant as positive events. In this way, explaining unfavorable events may lead to their integration into a meaningful higher-order narrative. However, some events are too deep in the unconscious mind and are thus too hard to integrate into a healthy narrative. People who suffer greatly from some kind of tragic event may be unable to integrate and attach meaning to them. In such cases, it is necessary to help the person to see a different explanation and imagine different lines of life, which will be perceived as different from the narrative breaker, and hence not as a break in the wholeness of self.

For example, often patients with post-traumatic stress disorder (PTSD) have difficulty living in the present [48]. Treatment of traumatic experiences needs to consider the way life has changed for the person experiencing trauma and how to interpret, explain, accept, and intertwine the traumatic happening into an overall and consistent line of life narrative. A traumatic event always damages human life to some degree; however, even so, a search for explanation and self-efficacy amid hardship can lead to resilience and growth [49]. This may be achieved by seeing one’s overall life story from a different perspective and integrating the trauma as part of a different story rather than as a break in the existing story. Using explanatory mechanisms and ideas from storytelling that connect memory with emotion, one can step outside the narrative and attach different responses to certain events to change the tone of the story. Nowadays we have the VR tools to create new stories that are immersive and convincing such that they become a visible line of life which can be reassessed and charged with different explanations and meanings.

### 2.3. The VR Experience for Rearranging the Life Narrative

**Figure 1:**
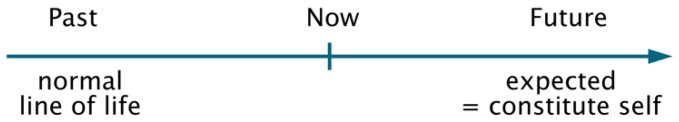
Normal line of line that helps to constitute the self.

VR visualization of a line of life can help someone understand the challenges in their self-narrative. A healthy perception of one’s life narrative helps someone to constitute a future self (Figure 1). In the case of a broken narrative (e.g., the loss of some ability due to a traumatic event), the self-image projected into the future is not as clear or robust as that of a healthy person [50,51,52]. VR offers a new perspective [53], and so, during immersion, one’s life vision can change. A vision of the future serves as a constitution of the self and a reassurance of one’s life story.

Some authors question whether there is an objective view at all and whether it is even possible to perceive reality in an unbiased way. This very broad topic, including the discussion of how VR may be a more objective medium for reassessing memories, is an issue for a separate study, particularly as it applies to trauma and worldview, and the authors are considering working on this in the future. Moreover, gaining a more objective perspective of past traumatic events is only one part of the proposed intervention here, as it is important to also discuss the idea of altering the flow of the VR memory reconstruction to develop new meaning, etc.

Indeed, VR can be designed in such a way that it is possible to identify if a person’s view of reality has been distorted or if memories have been altered by a traumatic event. For example, a person may share his or her view of an event and then when the event is recreated in a more consistent way in VR, using real data, it may add different perception to the memory (e.g., one can understand all the circumstances surrounding the event and release such feelings as self-blame and guilt). While the importance or severity of traumatic events should not be disregarded, a calm view may help the patient to process them faster [54]. An event that is seen, subjectively, as traumatic, is more likely to cause PTSD. VR can change strongly held views that such events were inevitable, are unchangeable, and have been utterly damaging [36]. Presumably, then the patient may be able to understand the memory without the immediate trauma of the event shaping their perceptions.

That is why VR experiences can be enhanced by scenarios which are different from reality and suggest novel ways to view the line of happenings in a life. A therapist, in the case of treatment of more serious conditions, may guide the storyline (the virtual experience) in order to obtain advanced therapeutic results. This intervention may be chosen in cases when a different mindset is necessary to improve cognitive disbalances (i.e., to achieve cognitive restructuring) [14] by, for example, changing someone’s perspective and giving them a more objective view of events (e.g., accepting inevitability and giving up guilt as in “It couldn’t be helped”), giving new interpretations (e.g., adding meaning such as “It was really a hard experience, but I survived and I am stronger now”), seeking resolution in conflicting situations (e.g., “Everyone has some kind of struggle in life that might have made them a bad person”), reassessing tense emotions that remain in memory (e.g., “I cannot hate him/her forever; I must forgive to feel free”), or detaching from personalized interpretations of events (e.g., “Such things always happen to me”, “I’m always having this kind of bad luck”). These specific examples of personal narratives shape a traumatized reality, and VR-based therapy can change these seemingly rigid realities into a healing state of mind.

### 2.4. Psychological Trauma as a Break in the Narrative Line

**Figure 2:**
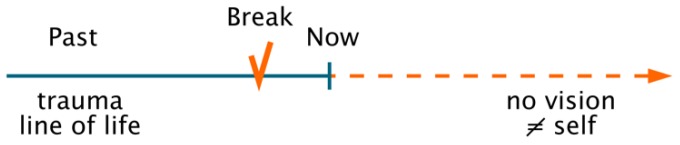
Traumatic experiences breaks the line of life and damage the self-concept.

As described, from a philosophical point of view, “[t]he narrative of a life can be one with multiple subplots, digressions, and deviations from the main narrative stream; it need not be linear in a simple-minded way” [20]. Therefore, in cases when unexpected events occur, the unity of human life [55] can be seen as broken [38]. The most severe conditions of broken self-narrative result from traumatic events (Figure 2), inducing, among other issues, a lack of future vision, distorted views of reality, and problems with self-identification. As memories are emotionally charged, they become substantial parts of the life story that someone uses to constitute their self.

However, therapy, in the context of self-narrative, can introduce an alternative narrative, or in philosophical and storytelling terms, a fiction that “works” [15] by creating meaning out of a traumatic event [38] and including it in a consistent way in the person’s life story.

In what way is this different from the above examples of cognitive restructuring techniques? When a person cannot envision his or her future self, or life with trauma, he or she must find ways to perceive the past events in a different way. For example, during exposure therapy, a PTSD patient re-experiences the events that resulted in the psychological trauma and builds resilience by reacting in a less emotional way to them. The proposal here is not to stop at this point, but to then provide experiences that are different from those that actually happened, and so give the patient a chance to experience some kind of transformation and cathartic healing. In many cases of trauma this might seem very difficult. However, in VR, imagining different plots within the story and acting in a different way may provide the person with a sense of control over their life and the ability to imagine a future with diverse outcomes. The opportunity to overcome negative events and to see a positive future would re-personalize the line of life and encourage steps toward creating that future. How would this be achieved in concrete and technical terms?

## 3. Proposal for Solving the Problem with the Line of Life

### 3.1. Addressing the Break in the Narrative Line

One way to address changes in the direction of one’s line of life is through comparison with other circumstances which were successfully integrated into one’s narrative line. Such innate ability can, however, be disrupted by events such as psychological trauma. Reconstructing a coherent story about one’s life and imagining a clear line of life, including a future image of self, depends on exercising one’s innate ability to internalize stories and their meanings. VR can support this ability; revisiting one’s life with the help of storytelling techniques, such as transformation, can lead to catharsis and growth.

Even though a VR narrative may be perceived as fiction, from a philosophical point of view [15], it can be modified, and hence serve as a cognitive restructuring story. When internalized, this can help a person, immersed in VR, to create a new self-narrative, thus seeing life in a new way in possible new directions. One can argue that someone cannot change the self. However, if the self is perceived as an “abstractum” ([15] p. 103), then the author of the narrative is partially removed from the story. Separating oneself from the narrative, which includes the challenging event, allows one to see events as part of a bigger picture. Exposure to VR can provide such perspectives.

**Figure 3:**
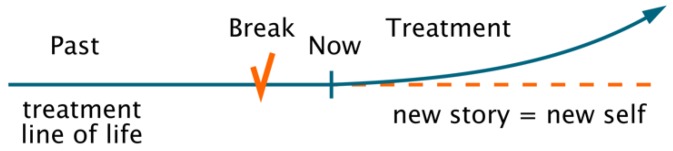
Treatment as creation of a new story of the self.

A conceptual analysis of the ways to address conflict in life can be used as therapeutic treatments. Even if we, and our stories, are merely constructions for keeping track of the history of the body, they can still be considered entities that have been developed in different stages of life, and so addressed in VR, with various positive results. For example, such a view of one’s life story could help a person suffering from trauma to step out of the traumatic story. This would allow the individual to identify less with his or her image of a broken self, re-create the past into a more meaningful story, and adopt a new explanatory paradigm or alternative line of life (Figure 3).

### 3.2. Detaching from the Self

Whether an individual’s self-narrative can be changed or not will determine the effectiveness of VR exposure. People’s associations with events may be more or less flexible (e.g., “I cannot change myself, that’s the way I am”).

Even if complete separation of the self-created in the life story is not possible, it is possible to realize that one’s traumatized self was influenced by particular events and is distinct from the self who created the prior life story. Such realization makes it possible for someone to make changes to the self in order to recover from these events. This could happen in experiences when one perceives his or her overall line of life with all the meanings and images of the self he or she has created thus far.

People are always able to change things about themselves, even the way they perceive their past or present, and so reconstitute the future self. Acknowledgement of authorship of one’s story can be achieved through self-reflection. Experience of one’s life story in VR can encourage self-reflection through observing one’s own image [20,56] or line of life. This may elicit understanding of why something is causing pain, how one has interpreted past events, and what one is prone to believe about one’s life story. In turn, this may lead to someone realizing that, for example, they are more sensitive than others to certain stimuli. They may be more prone to react strongly to certain events and so experience a break in their self-narrative. A VR experience can aid cognitive restructuring and recreation of self, and the narrative that constitutes that self, through representing one’s line of life again.

Restoring one’s storytelling ability, as an author who controls the line of the life narrative, would also restore the strength one has lost during a weakening experience such as a traumatic event. Such an ability to influence the narrative that constitutes the self, which everyone possesses and understands, can reach to a very deep level. Therefore, as in Figure 3, people have the capacity to tell a different personal story or retell their own story in a different way with added meaning or explanation.

## 4. Effects of the Resolution on the Line of Life and Proposal for Treatment Design

### 4.1. Authoring the Experience of Line of Life in VR

The VR exposure involves creating a new story of the self that is visualized as an alternative line of life. Reconstructing and internalizing a new story in VR is anticipated to be an effective and fast way towards recovery from psychological trauma as it complements the ability of human beings to tell an imaginative story. A flexible way of changing the story in VR, while treating a patient with traumatic disorder, can be especially effective, as this allows the patient to immediately respond.

Immediate responses reinforce a patient’s sense of being an author, forming his or her own life story to date prior to the traumatic event. This would then give the patient the sense of also being the author of the traumatic experience, at least in terms of interpretation and inclusion in the overall line of life. Overviewing the story as something linear and happening in the VR helps someone to detach the events from real life and to see everything as a narrative that can be reviewed and even controlled in its effect on the overall life narrative.

Enabling the storytelling ability of the patient would help him or her make an important realization, namely thoughts becoming narratives that in turn become beliefs and shape reality. Understanding that memory is colored by personal interpretation and choosing what to view can help someone see progress in a situation that seemed initially to be a loss.

### 4.2. Designing VR Experience to Create New Meaning and Explanation

Possible steps in workflow design for line of life visualization and creation of a new line that would help constitute the future self and aid healing are suggested below. However, as this study is not able to provide empirical results, it serves as a development in interdisciplinary theory and suggests the way that gradually adapted scenarios can serve the above purpose. In the future, this broad design may be realized and complemented by more concrete and technical design ideas.

Therefore, what could be the exact mechanisms for creating the life story of a person in VR? VR experience can reinforce awareness that one’s storyline or line of life are always changing with new lines emerging (Figure 3). A person has the chance to interpret and revisit the line of life through gradual and repeated exposures, and to take ownership of the story; told in a new way as a new line of life. Any kind of event can make sense when seen within a new narrative framework, making it one’s personal story, or part of a quest for a personal line of life.

When one’s life story takes an undesirable direction, seeing this event from a higher perspective might even offer insights to the self to help one grow. VR can assist people by reminding them of their narrative capability, helping them to regain control over their life story presented as a line of life.

A design for the experience of one’s line of life in VR is proposed, considering the following ideas:

⚬ design an experience of reliving/retelling one’s line of life,
⚬ change the point of view about certain events to gain a healthier perspective of them,
⚬ perform cognitive restructuring of certain events that lead to a healthier and more positive viewpoint,
⚬ create personalized interpretations of certain events from the life narrative,
⚬ achieve authoring and gain control over their life story,
⚬ redesign the line of life to promote a healthier self.

**Figure 4:**
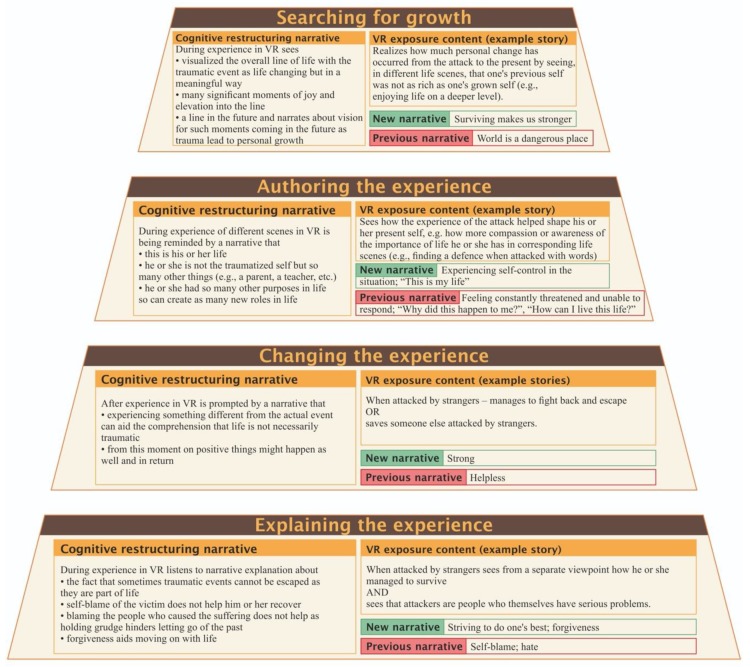
Procedure to reexperience one’s line of life under the guidance of a therapist in the case of physical assault.

The following concrete suggestion for workflow design involves the presence of a therapist. A design without a therapist should be considered more carefully and is therefore the subject of a separate study. In order to achieve the best outcome from the experience, all the functions in Figure 4 should be performed. The first two can, however, change places.

This example workflow, relating to a serious case of trauma after physical attack, should only be applied after robust exposure treatment and cognitive restructuring.

Changing self-image from weak and helpless to that of a survivor is the first step. The second step includes avoidance of negative thought patterns that hinder letting go and moving on, such as self-blame, and turning them into a realization that one has done one’s best in the given situation; and also, that that blame, whether it is self-blame or the blame of others, does not help in the healing process. Next is authoring the experience; realizing that one has done so many other things that were positive and growing experiences will promote wholeness of view toward life and release from the traumatic event. Searching for growth will conclude the process by provoking a survivalist stance toward life’s adversities. Through this procedure, one can take control of one’s narrative about the past and develop healthier meanings to integrate into the overall life story. Moreover, through the VR experience, one may be able to construct a new self-narrative and restore a healthy vision of the future self.

## 5. Final Remarks

This paper proposes that storytelling and memory restructuring can promote healthier and more positive outlooks on life. The VR medium allows reconstruction of one’s line of life, so it provides an empowering experience that returns ownership of one’s narrative self. This thinking can be applied to the practical design of immersive VR environments that aim at behavioral change, health improvement, self-development, and so on.

## 6. Ethics Statement

The study did not involve human experimentation or data, and therefore did not require approval of an Ethics Committee.
